# Co-circulation of multiple subtypes of enterovirus A71 (EV- A71) genotype C, including novel recombinants characterised by use of whole genome sequencing (WGS), Denmark 2016

**DOI:** 10.2807/1560-7917.ES.2017.22.26.30565

**Published:** 2017-06-29

**Authors:** Sofie E Midgley, Astrid G Nielsen, Ramona Trebbien, Mille W. Poulsen, Peter H Andersen, Thea K Fischer

**Affiliations:** 1 Section for Virus Surveillance and Research, Statens Serum Institut, Copenhagen, Denmark; 2 Infectious Disease Epidemiology, Statens Serum Institut, Copenhagen, Denmark; 3 Center for Global Health, Department of Infectious Diseases, University of Southern Denmark, Odense, Denmark

**Keywords:** enterovirus, classification, Diagnostics, whole genome sequencing, EV-A71, WGS, NGS, next generation sequencing

## Abstract

In Europe, enterovirus A71 (EV-A71) has primarily been associated with sporadic cases of neurological disease. The recent emergence of new genotypes and larger outbreaks with severely ill patients demonstrates a potential for the spread of new, highly pathogenic EV-A71 strains. Detection and characterisation of these new emerging EV variants is challenging as standard EV assays may not be adequate, necessitating the use of whole genome analysis.

Enterovirus A71 (EV-A71) has been detected in Denmark in relatively low numbers since 2001. Of the different genotypes of EV-A71, only B5, C1, C2, and C4 have been identified [1,2]. All genotypes have been either associated with neurological symptoms, or milder hand, foot, and mouth disease (HFMD) in Denmark. EV-A71 subtype C4a, commonly circulating in Asia, has been identified as the cause of extensive outbreaks of HFMD with neurological complications and fatalities [3,4]. During a pilot project validating next generation sequencing (NGS) technology for full genome typing of EVs at the National World Health Organization (WHO) Reference Laboratory for Poliovirus at Statens Serum Institut (SSI), a novel EV-A71 variant was identified. This variant was shown to be genetically closely related to a recently published novel EV-A71 genotype C1 recombinant variant identified in Germany [5,6]. Re-analysis of sequence data and full genome re-sequencing of selected samples from the Danish EV surveillance database revealed additional viruses phylogenetically closely related to this new variant.

## Laboratory analyses

Samples included in the analysis were collected through the Danish EV surveillance system [7]. They were genotyped using VP2 and/or VP1 PCR assays [8,9] and sequenced as described previously [2]. All 20 EV-A71 cases from 2016, as well as additional 12 historical cases previously subtyped as C1 were analysed. NGS was carried out directly on clinical sample material, as described previously [10], for a subset of samples (n = 10) and one sample was characterised from cultured material obtained as part of another study. The Illumina MiSeq platform was used to generate the sequencing data that were analysed using the CLC genomics workbench. Consensus sequences were exported as fasta files, and aligned with all available full-length EV-A71 genomes downloaded from the National Center for Biotechnology Information (NCBI) GenBank using SSE v1.3[11]. Phylogenetic analysis was carried out using MEGA 6 [12], maximum likelihood with a general time reversible model, gamma distribution and invariable sites, and 1,000 bootstrap replications. Bootscanning analysis was also carried out using SimPlot v3.5.1 [13]. Sequences have been submitted to GenBank, accession numbers pending.

## Enterovirus A71 genotype C cases 2001–2016

The most common sample material available for the 32 EV-71 RNA-positive patients was stool (n = 22) followed by vesicular fluid (n = 6). Other sample materials were urine, cerebrospinal fluid (CSF), respiratory secretion and biopsy. (Table 1).

**Table 1:** Clinical description of enterovirus A71 genotype C cases, Denmark, 2001–2016 (n = 32)

Year of detection–case ID	Age	Sample material	Sequence type^a^	EV-A71 genotype	Symptoms	Hospital admission
01–01	1–2 years	Unknown	VP2	C1	Guillain–Barré syndrome	Unknown
07–01	5–10 years	Vesicular fluid	VP2,WGS	C6	Vesicles	Unknown
07–02	< 6 months	Stool	VP1,VP2,WGS	C1	Unknown	Unknown
07–03	< 6 months	Stool	VP1,VP2,WGS	C6	Unknown	Unknown
07–04	1–2 years	Stool	VP1,VP2,WGS	C1	Diarrhoea for 3 weeks after travelling	Unknown
07–05	< 6 months	Stool and urine	VP1,VP2	C1	Meningitis, fever, abdominal pain	Unknown
10–01	35–40 years	Vesicular fluid	VP1,VP2	C1	HFMD– rash, vesicles	< 1 day
10–02	0–2 months	Stool	VP2	C1	Sepsis – fever > 38.5, diarrhoea, vomiting blood	5 days
10–03	0–2 months	Stool and CSF	VP1,VP2	C1	Meningitis	Unknown
14–01	25–30 years	Vesicular fluid	VP1,VP2	C1	HFMD – rash, vesicles	No
14–02	1–2 years	Respiratory secretion	VP2	C1	Respiratory symptoms	Unknown
14–03	< 6 months	Unknown	VP1,VP2,WGS	C6	Fever > 38.5 °C, diarrhoea, vomiting	1 day
16–01	< 6 months	Small intestine biopsy	VP1	C4	Sudden death, also *Clostridium difficile* infection	Unknown
16–02	< 6 months	Stool	VP1,VP2,WGS	C6	Fever > 38.5 °C, diarrhoea, rash	1 day
16–03	1–2 years	Vesicular fluid	VP2,WGS	C6	HFMD – rash, vesicles	no
16–04	< 6 months	Stool	VP1,VP2,WGS	C6	Fever > 38.5 °C, diarrhoea, rash	< 1 day
16–05	6–11 months	Stool	VP1,VP2,WGS	C6	HFMD – fever > 38.5 °C, rash, vesicles, vomiting	6 days
16–06	< 6 months	Stool	VP1,VP2,WGS	C new/C6 ^b^	HFMD – fever > 38.5 °C, rash, vesicles, respiratory symptoms	4 days
16–07	< 6 months	Stool	VP2	C1	Encephalitis – fever > 38.5 °C, abnormal sensitivity to stimuli, diarrhoea, rash, respiratory symptoms, sepsis-like symptoms	5 days
16–08	4–5 years	Stool	VP2,WGS	C new/C6 ^b^	Meningitis –neck stiffness, affected consciousness, fever, rash, vomiting	5 days
16–09	< 6 months	Stool	VP1,VP2	C1	Unknown	Unknown
16–10	1–2 years	Stool	VP1	C1	Unknown	Unknown
16–11	1–2 years	Stool	VP2	C1/C6 ^c^	Fever, rash, vesicles, diarrhoea	Unknown
16–12	< 6 months	Stool	VP1	C1	Fever > 38.5 °C, rash	4 days
16–13	< 6 months	Stool	VP1,VP2	C1	Unknown	Unknown
16–14	< 6 months	Stool	VP2	C1/C6 ^c^	Fever > 38.5 °C, group B *Streptococcus* sepsis	10 days
16–15	< 6 months	Stool	VP2	C1	Fever	7 days
16–16	< 6 months	Stool	VP1	C6	Fever > 38.5 °C, vomiting, sepsis	4 days
16–17	< 6 months	Stool	VP2	C1/C6 ^c^	Unknown	Unknown
16–18	1–2 years	Vesicular fluid	VP2	C1	Unknown	Unknown
16–19	25–30 years	Vesicular fluid	VP2	C1	Unknown	Unknown
16–20	1–2 years	Stool	VP2	C2	Unknown	Unknown

HFMD: hand, foot, and mouth disease.

^a^ VP1: sequence was obtained from VP1 PCR; VP2: sequence was obtained from VP2 PCR; WGS: sequence was obtained from whole genome sequencing.

^b^ Grouping with C6 in near-complete genome analysis, but not with C6 or C1 in VP1 analysis. ^c^ Grouping with C6 when analysing the VP1 region, but no complete genome sequence available and therefore not possible to confidently assign as C6.

Patient ages ranged from 7 days to 36 years with a median of 3 months and inter-quartile ranges of 2 and 12 months, respectively, and 11 of 32 patients were females. Clinical information was available for 23 of 32 cases, collected as part of the enhanced EV surveillance system in place [7]. Six cases had HFMD, 11 had gastrointestinal symptoms, 14 had fever, five had central nervous system involvement (Guillain–Barré syndrome, meningitis, encephalitis), and there was one death. The death and one of the sepsis cases were associated with a bacterial co-infection (*Clostridium difficile* and group B *Streptococcus*, respectively). Hospital admission data was available for 13 cases, and the hospitalisation ranged from less than one day to 10 days (average 3.6 days, SD 2.8).

## Phylogenetic analysis

**Figure 1:**
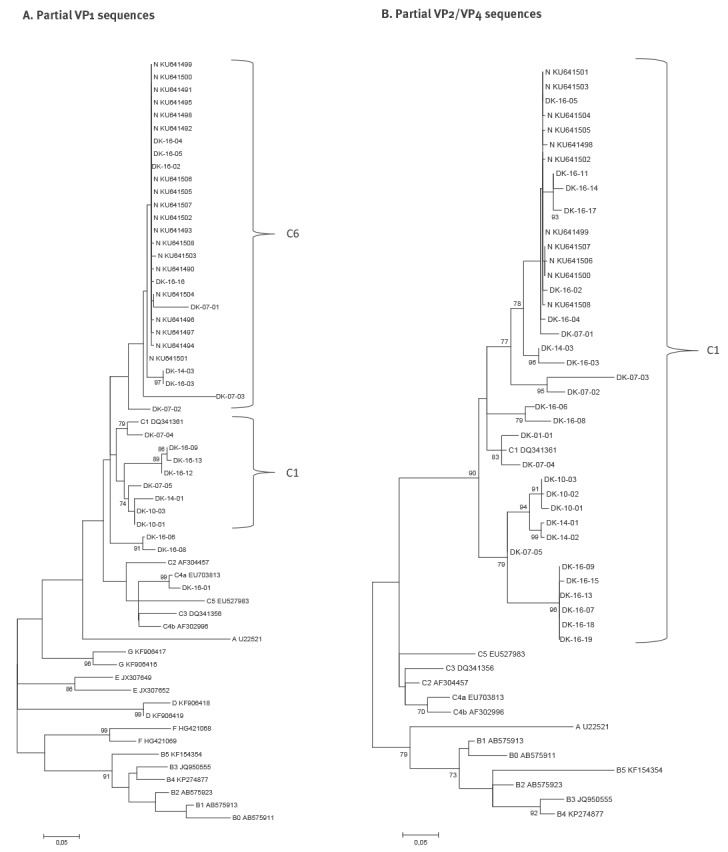
Phylogenetic analysis of enterovirus A71 genotype C cases, Denmark, 2001–2016

**Figure 2:**
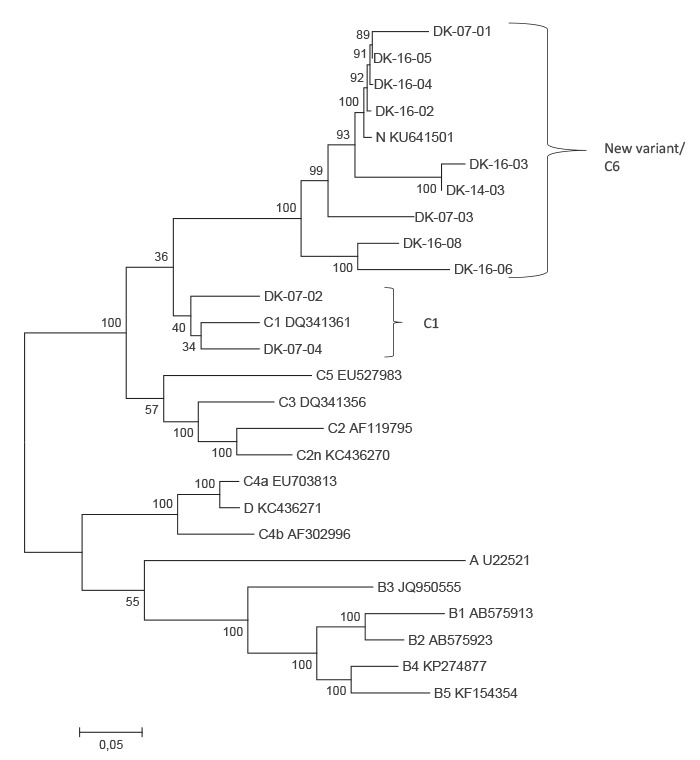
Phylogenetic analysis of near-complete genome sequences, enterovirus A71 genotype C cases, Denmark, 2001–2016

Phylogenetic analysis of partial VP2/VP4 and /or VP1 sequence data showed that 17 samples could be characterised as belonging to EV-A71 genotype C1 and 13 samples were closer to a new variant recently identified in our laboratory. Moreover, one EV-A71 genotype C2 and one C4 were identified (Figure 1). NGS data was obtained for a total of 11 strains, nine were found to belong to the new genotype, and two were C1 (Figure 2).

German new variant strains are identified with a capital N as a prefix, Danish strains are identified by the case ID as described in Table 1 prefixed by DK. Genotypes C1 and C6 are marked with brackets. Reference sequences for other genotypes are prefixed by the genotype, followed by the GenBank accession number.

Phylogenetic analysis was carried out using MEGA 6 [12], maximum likelihood with a general time reversible model, gamma distribution and invariable sites and 1,000 bootstrap replications. The bootstrap support is indicated at nodes, showing only values of > 70%. The bar denotes 0.05 nt substitutions per site.

German new variant strains are identified with a capital N as a prefix, Danish strains are identified by the case ID as described in Table 1 prefixed by DK. Genotypes C1 and C6 are marked with brackets. Reference sequences for other genotypes are prefixed by the genotype, followed by the GenBank accession number.

Phylogenetic analysis was carried out using MEGA 6 [12], maximum likelihood with a general time reversible model, gamma distribution and invariable sites, and 1,000 bootstrap replications. The bootstrap support is indicated at nodes. The bar denotes 0.05 nt substitutions per site.

In the VP1 and near-full genome phylogenetic analyses, the new variant forms a clade separately from other C1 viruses. The new recombinant strain was identified in a sample dating back to 2007. In the WGS three clades were seen: (i) one containing Danish strains from 2007 and 2016 as well as strains from Germany, (ii) one with single Danish strains from 2014 and 2016, and (iii) one with two Danish strains from 2016. There was no difference between the genotypes regarding the clinical symptoms of the cases (Table 1). In the VP2 analysis it was not possible to clearly distinguish between C1 and the new variant.

**Figure 3:**
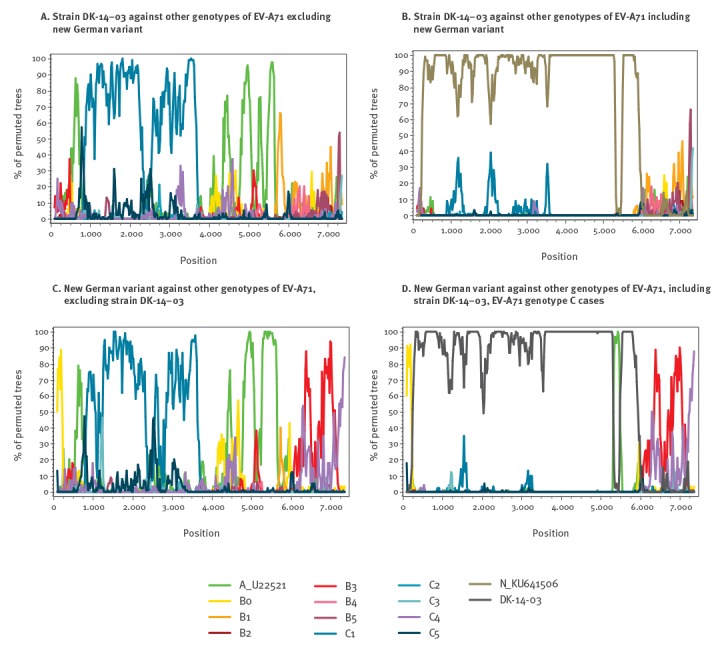
Bootscanning and SimPlot analysis of novel enterovirus A71 genotype C cases DK-14–03 (Denmark 2014) and KU641506 (Germany 2015)

DK: Denmark; EV: enterovirus.

Bootscanning analysis showed that Danish new variants were nearly identical to the German new variants over the majority of the genome. However, there was no similarity in the 3D^pol^ region, where both the German and the Danish strains differed from all published subtypes (Figure 3). The German strains appear to have a mosaic genome, more closely related to different genotypes in different parts of the genome. When analysed without comparison to the German strain, the Danish variant also showed a similar mosaic genome (Figure 3). Both strains appear to be closest to C1 in the VP1 region of the genome.

## Discussion and conclusions

This study documents the circulation of new emerging EV-A71 genotype C variants associated with neurologic symptoms, as well as HFMD, in the Danish population. Novel EV-A71 variants were detected with WGS, and could be traced back to the first detection in the Danish population in 2007. Phylogenetically, a higher level of genetic variation was seen among the Danish strains as compared with the previously reported German EV-A71 C1 variant strains [5,6] suggesting that these novel variants have arisen from genetic reassortment of EV-A71 viruses over a prolonged period of time.

Three EV-A71 C variant clades were identified in Denmark one of which contains Danish EV-A71 C strains from 2007 and 2016 as well as the German 2015 strains. The phylogenetic analyses in this study show that the new variant EV-A71 sub-genotype C viruses form a clade separate from the C1 viruses, and the authors propose that these new variants are seen as a new genotype, C6, rather than a lineage of C1.

Both German and Danish new variants appear to be recombinant forms, with 3D^pol^ regions of separate origins. This was previously described for the German strains [6]. In fact, both the German and Danish strains appear to be mosaic, a result of several recombination events throughout the genome. The co-circulation of multiple genotypes of EV-A71 in one country during a single season/year, as demonstrated in this study, provides the environment for the appearance of future novel recombinant variants.

EV-A71 genotype C4 subtypes associated with more severe clinical outcomes than other EV-A71 genotypes and subtypes have previously been described [3,4]. The new C variant was described as emerging in 2015 in Germany and associated with rhomboencephalitis/brainstem encephalitis and severe neurological and cardiopulmonary complications [5,6,14]. However, the collection of samples for the EV surveillance system, as in the case of Denmark and Germany, may introduce a bias in this regard. The new EV-A71 genotype C variant identified in Denmark in 2016, was associated with both neurological symptoms and HFMD, illustrating the ability of EVs to cause a wide range of symptoms with rare cases of severe complications.

New emerging EVs have already demonstrated their potential to cause devastating epidemics such as the major EV-A71 epidemics in Asia and South Pacific Region. There is therefore a need to detect and monitor these viruses closely. In addition to detection and reporting of an emerging new EV-A71 recombinant virus, a proposed genotype C6, this study demonstrates important challenges in detection as well as characterisation of emerging EV infections. Current state-of-the-art EV PCR-based methods continuously need to undergo evaluation to ensure that primers for diagnostics, as well as typing, maintain the ability to detect and fully classify new EV variants beyond the (sero)type level. PCR-based typing relies on the amplification of short genome fragments, and as a consequence may not only result in missed detection of new genotypes, but also in misclassification due to a lack of appropriate reference sequences. Furthermore, timely and public sharing of whole EV genome sequence data are essential for detection of new variants.

The possible severity of EV-A71 infections together with the continuing evolution and appearance of new EV-A71 genotypes, as well as other emerging EV causing neurological disease, underscores the importance and relevance to prioritise strengthening of EV surveillance globally.
